# Mechanosensitive adhesion G protein-coupled receptors: Insights from health and disease

**DOI:** 10.1016/j.gendis.2024.101267

**Published:** 2024-03-16

**Authors:** Shiying Sun, Wen Wang

**Affiliations:** a Department of Orthodontics, School and Hospital of Stomatology, Hebei Medical University, Shijiazhuang, Hebei 050017, China; b Hebei Key Laboratory of Stomatology, School and Hospital of Stomatology, Hebei Medical University, Shijiazhuang, Hebei 050017, China; c Hebei Clinical Research Center for Oral Diseases, School and Hospital of Stomatology, Hebei Medical University, Shijiazhuang, Hebei 050017, China

**Keywords:** Activation mechanism, aGPCR, Health and disease, Mechanotransduction, Signaling pathway

## Abstract

Ontogeny cannot be separated from mechanical forces. Cells are continuously subjected to different types of mechanical stimuli that convert into intracellular signals through mechanotransduction. As a member of the G protein-coupled receptor superfamily, adhesion G protein-coupled receptors (aGPCRs) have attracted extensive attention due to their unique extracellular domain and adhesion properties. In the past few decades, increasing evidence has indicated that sensing mechanical stimuli may be one of the main physiological activities of aGPCRs. Here, we review the general structure and activation mechanisms of these receptors and highlight the lesion manifestations relevant to each mechanosensitive aGPCR.

## Introduction

Mechanical forces are essential for the embryonic development of living organisms, as well as for multiple physiological and pathological processes. Cells continuously undergo diverse mechanical messages, such as tensile, compressive, and shear stresses. Cell function is additionally regulated by the rigidity or elastic modulus of the extracellular matrix.^1^ A process known as mechanotransduction is needed to translate those mechanical stimuli into biochemical signals, which in turn regulate how cells behave. There are three phases in this process: i) mechanotransmission: mechanical forces are transmitted to mechanosensitive receptors; ii) mechanosensing: mechanosensitive macromolecules undergo conformational changes, and then the new conformation triggers downstream signaling events; and iii) mechanoresponse: cells respond to perceived mechanical signals through complex cellular signaling networks.^2^^,^^3^

Several proteins function as mechanosensitive receptors, including ion channels, cell surface receptors (*e.g.*, integrins and cadherins), and the G protein-coupled receptors (GPCRs).^4^^,^^5^ The idea that ion channels and cell surface receptors can act as mechanosensitive receptors has long been widely accepted. In the case of GPCRs, they were previously thought to be chemoreceptors, however, several studies have revealed that they are also capable of sensing mechanical stresses. Due to their dual chemoreceptive and mechanoreceptive features, GPCRs have gained much attention as polymodal sensors. GPCRs are a group of membrane proteins with a seven-transmembrane (7TM) domain, so-called due to their capacity to bind to G proteins and then regulate downstream cascades. According to the GRAFS classification system, glutamate, rhodopsin, adhesion, frizzled/taste 2, and secretin are the five primary groups that make up the GPCR superfamily.^6^ As the second largest member of the GPCR superfamily, adhesion G protein-coupled receptors (aGPCRs) were once considered to belong to the secretin family, but they have recently been identified as a distinct family due to their unique autoproteolysis and long N-terminal domains. aGPCRs can be further subdivided into nine subfamilies, and each aGPCR is given a name beginning with the prefix “ADGR” with a corresponding letter and number.^7^ Aside from their role in basic physiological processes like cell adhesion and migration, aGPCRs have a strong relationship with functional regulation and disease progression in many different systems. Some of these aGPCRs can perform their mechanosensory functions in the presence of mechanical forces. They sense mechanical stimuli and transmit them into the cells through specific signaling cascades, initiating the corresponding physiological or pathological processes.^8^ Furthermore, these mechanosensitive aGPCRs have been shown to correlate with other diseases. Nevertheless, the existence of mechanotransduction processes in those diseases remains to be further explored.

In this review, we sum up the structural features, activation modes, and signaling of aGPCRs and discuss the physiology and pathology of mechanosensitive aGPCRs.

## General characteristics of aGPCRs

**Figure 1:**
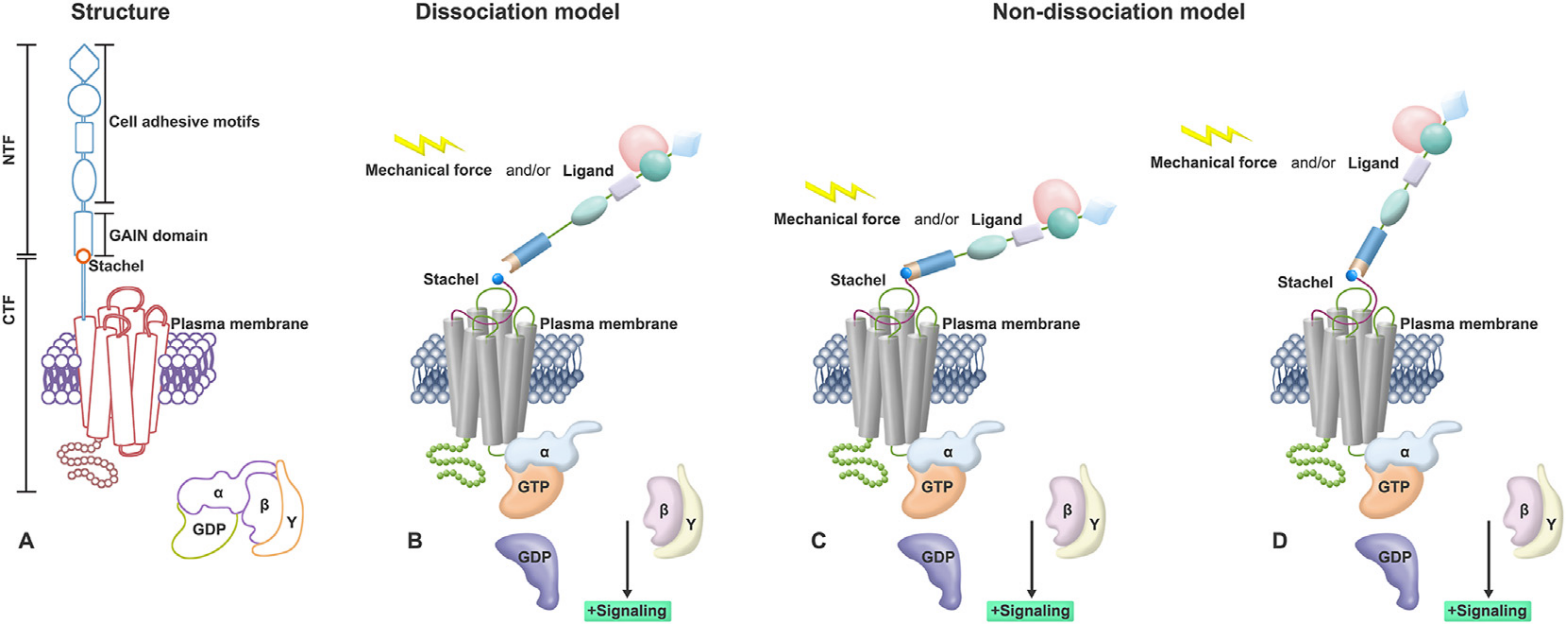
Structure and the tethered activation mechanism of aGPCRs. **(A)** The basic structure of aGPCRs. The NTF contains cell adhesion motifs and a large part of the cleaved GAIN domain. The CTF contains the Stachel sequence, the 7TM domain, and the intracellular C-terminus. **(B)** The dissociation activation model of aGPCRs. Ligands and/or mechanical forces induce the shedding of the NTF and the exposure of the Stachel sequence, thereby activating downstream signals. **(C, D)** The non-dissociation activation model of aGPCRs. The position (C) or conformation (D) of the GAIN domain changes in response to ligands and/or mechanical forces, which is followed by interaction of the ECD with the 7TM (C) or exposure of the Stachel sequence through a surface opening in the GAIN domain (D). aGPCRs, adhesion G protein-coupled receptors; CTF, C-terminal fragment; ECD, extracellular domain; GAIN, GPCR autoproteolysis-inducing; NTF, N-terminal fragment.

The basic structure of aGPCRs includes a GPCR autoproteolysis-inducing (GAIN) domain, an extracellular N-terminal domain, a 7TM domain, and an intracellular C-terminus (Fig. 1A). The GAIN domain contains a GPCR proteolysis site (GPS). aGPCRs can self-cleave at this site and produce the following two noncovalently associated fragments: the N-terminal fragment (NTF) and the C-terminal fragment (CTF).^9^ The NTF, which mainly serves as an adhesion agent, consists of a large part of the cleaved GAIN domain and an extracellular adhesion protein domain formed by different motifs. The CTF, which is responsible for binding to G proteins, is composed of a fraction of the proteolyzed GAIN domain, a linker region connecting the GAIN domain to transmembrane helix 1, the 7TM domain, and the entire intracellular domain. The portion of the proteolyzed GAIN domain and the linker region are collectively known as the “Stachel sequence”.^10^^,^^11^ Autoproteolysis (also known as cleavage) usually occurs spontaneously during a time when the receptor is transported to the plasma membrane, and this process is ligand-independent. However, the binding of ligands may separate NTF and CTF produced by cleavage.^12^ Notably, several studies have shown that GPS cannot mediate autoproteolysis alone and can only function within the intact GAIN domain.^13^^,^^14^ Moreover, aGPCRs lacking the GAIN domain (*e.g.*, ADGRA1) and aGPCRs containing variant GPS motifs (*e.g.*, ADGRF2 and ADGRF4) cannot be autoproteolyzed.^15^^,^^16^ Consequently, intact GAIN domain and specific GPS motifs are necessary for autoproteolysis.

It is now widely accepted that aGPCRs function mainly through a tethered activation mechanism, which means the large N-terminus of the receptors acts as a tether to transmit mechanical signals into the cell. In this mechanism, the Stachel sequence can be further divided into two functional units. Its N-terminal agonistic portion, which is made up of the first seven amino acids, serves as an intramolecular tethered agonist (TA). Meanwhile, the C-terminal half ensures correct targeting of the TA to the binding pocket of the 7TM.^17^ A noncovalently linked extracellular domain (ECD) generally inhibits the 7TM domain of the receptor. Suppression is achieved by encrypting the TA, which, when exposed, exerts its agonist effect.^18^ Recently, researchers have proposed two main models for the Stachel sequence-mediated activation mechanism. The first is the dissociation model, also termed the shedding or the one-and-done model. Engagement with ligands and/or mechanical stimulation induces dissociation of the NTF and the CTF, which exposes the TA and transforms the receptor's state from inactive to active. Autoproteolysis is a prerequisite for dissociation, and once dissociation occurs, it is almost impossible to re-encrypt the TA to terminate signaling (Fig. 1B).^19^^,^^20^ The second model is the non-dissociation model, also known as the displacement or tunable model. The location or conformation of the GAIN domain is altered by the binding of extracellular ligands and/or the application of mechanical forces. This is followed by transient interaction of the ECD with the 7TM (Fig. 1C) or exposure of the TA through a surface opening in the GAIN domain (Fig. 1D), which then activates downstream signals. This mode of regulation is reversible, and both receptor hydrolysis and division are dispensable for its activation.^21^^,^^22^ These two models are compatible with the view that aGPCRs can act as mechanoreceptors.

Numerous articles have emphasized the significance of the Stachel sequence in aGPCR activation, but the mechanism by which it interacts with 7TM remains unclear. Recent investigations have provided some tenable interpretations based on the cryogenic electron microscopy structures of the receptors. The undissociated GAIN domain protrudes flexibly into the extracellular space, which permits the ECD to bind to ligands there and keeps the encrypted TA away from the 7TM bundle.^23^ At this point, the Stachel sequence is arranged in a β-sheet configuration. However, after being released from the GAIN domain, it isomerizes into a horizontal U shape, which is divided into a hydrophilic segment (upper part) and a hydrophobic segment (lower part) along the bottom-opening of the U shape. The hydrophobic fragment's five amino acid residues are organized in a finger-like structure that contacts the hydrophobic pocket of the transmembrane domain. This hydrophobic interaction is necessary for the receptor to be activated by the Stachel sequence.^24^^,^^25^

As previously described, G proteins function as downstream signal transduction effectors of aGPCRs after they have been activated. In canonical G protein-dependent signaling, agonist occupancy induces receptor remodeling as a guanine nucleotide exchange factor (GEF) for G protein. GEF can catalyze the exchange of GDP for GTP on the Gα subunit and the dissociation of Gα from the Gβγ subunits. It results in the conversion of Gα from an inactive GDP-binding conformation to an active GTP-binding conformation.^26^^,^^27^ The signal is terminated when GTP is hydrolyzed to GDP.^28^ There are four different isoforms of G proteins (including G_s_, G_i/o_, G_q/11_, and G_12/13_). Activated G proteins enable the stimulation of distinct signaling pathways through corresponding secondary messengers.^29^ In most cases, G_s_ and G_i_ activate and inhibit adenylyl cyclase, respectively. G_q_ primarily activates phospholipase C, while G_12/13_ typically regulates small GTP-binding proteins.^30^ Some aGPCRs are well accepted to bind to signaling proteins other than G proteins and mediate intracellular signaling. β-Arrestin is a common effector in the G protein-independent signaling pathway.^31^ Upon recognition of activated aGPCRs by G protein-coupled receptor kinases, specific regions of the third intracellular loop and carboxyl terminus of the receptors are phosphorylated, which is followed by interaction of those receptors with β-arrestins. As a result, G protein activation is inhibited, non-G protein signaling is initiated, and receptor endocytosis through clathrin-coated pits is facilitated.^32^^,^^33^ In addition to β-arrestins, other effectors are also part of the G protein-independent signaling pathway. For instance, ELMO and Dock180 act together as a GEF for the small GTPase Rac. They activate Rac signaling after shaping into a trimeric complex with BAI1, thereby facilitating the engulfment of apoptotic cells.^34^ In conclusion, the unique physiological structures and activation processes of aGPCRs determine the intricacy of their signaling network and the diversity of their roles.

## Activation of mechanosensitive aGPCRs in different tissues

Based on the activation mechanism of aGPCRs mentioned in the previous section, it is clear that mechanical force can be engaged in the activation of aGPCRs. In addition, mounting evidence suggests that perceiving mechanical stimuli may even be one of the main physiological roles of aGPCRs. Currently, at least ten aGPCRs are known to be capable of detecting mechanical forces. Each of them will be discussed below according to different activation models (Table 1).

**Table 1:** Summary of the activation models of mechanosensitive adhesion G protein-coupled receptors (aGPCRs).

Mechanosensitive aGPCR	Activation model	Ligand	Mechanical stimulus	Tissue, cell, or structure involved
ADGRE2/EMR2	Dissociation	Dermatan sulfate, monoclonal antibody	Vibration	Mast cell
	Unknown	Complement factor H-related protein 1, monoclonal antibody	Cross-linking	Monocyte
ADGRE5/CD97	Dissociation	CD55	Shear stress	Conventional dendritic cell
	Dissociation	CD55	Shear stress	Leukocyte
ADGRG6/GPR126	Dissociation	Laminin-211	Cross-linking	Schwann cell
	Dissociation	Type IV collagen	Pushing force	Schwann cell
ADGRF5/GPR116	Dissociation	Surfactant protein D	Stretching	Alveolar type II epithelial cell
ADGRV1/GPR98	Dissociation	/	Shear stress	Focal adhesion
ADGRG5/GPR114	Non-dissociation	/	Vibration	/
ADGRL1-ADGRL3/CIRL1-CIRL3/Latrophilins	Non-dissociation	/	Touch, sound, proprioception	Chordotonal organ
	Unknown	/	Pain	C4da neuron
ADGRG1/GPR56	Dissociation	Collagen	Shear stress	Platelet
	Dissociation	Type III collagen	Cross-linking	Neuron
	Dissociation	Laminin-211, transglutaminase 2	Cross-linking	Oligodendrocyte precursor cell
	Dissociation	CG4, monoclonal antibody	Cross-linking	Melanoma cell
	Unknown	Type III collagen	Mechanical load	Muscle
	Non-dissociation	Synthetic ligand	/	/

### Model of activation mainly through dissociation

#### ADGRE2/EMR2

ADGRE2/EMR2 belongs to the ADGRE subfamily. Members of this subfamily contain epidermal growth factor (EGF)-like domains in their ECDs and are therefore known as EGF-TM7 receptors.^35^ The receptor is most abundantly expressed in granulocytes and monocytes/macrophages of the immune system.^36^ Notably, the fourth EGF domain of EMR2 is highly conserved and can mediate specific binding of the receptor to its endogenous ligand dermatan sulfate (DS).^37^ Lin et al showed that EMR2 could undergo autoproteolytic cleavage at the GPS, and this finding provided a paradigm for exploring the hydrolysis mechanisms of other GPS motif-containing proteins.^38^

**Figure 2:**
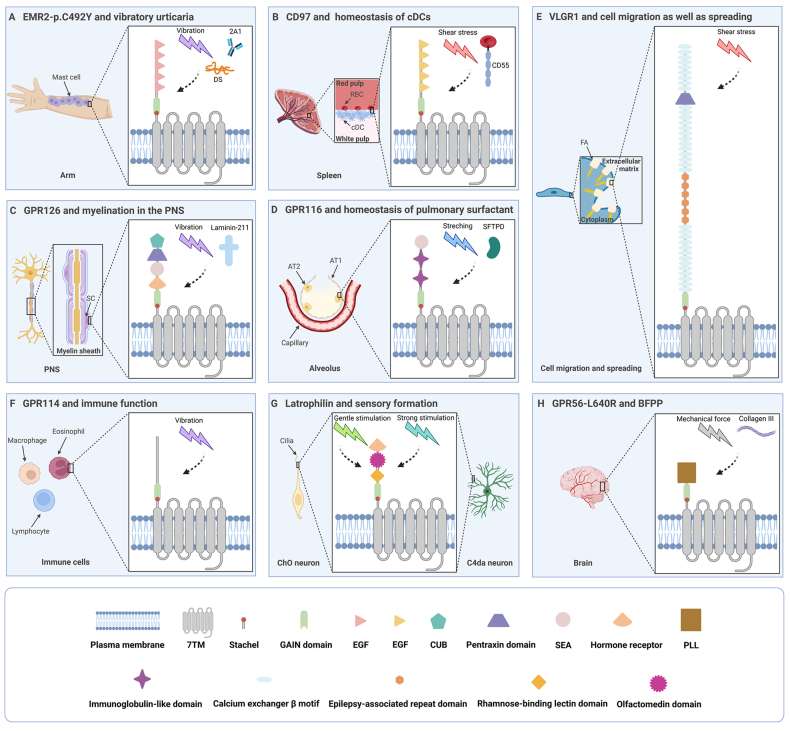
Structures of mechanosensitive aGPCRs and their participation in typical physiological and pathological processes (Created with BioRender.com). **(A)** The missense variant in EMR2, p. C492Y, is associated with the development of vibratory urticaria. **(B)** CD97 maintains the homeostasis of cDCs. **(C)** GPR126 is related to myelination in the PNS. **(D)** GPR116 maintains the homeostasis of pulmonary surfactant. **(E)** VLGR1 regulates cell migration and spreading. **(F)** GPR114 is involved in the immune function. **(G)** Latrophilin modulates sensory formation. **(H)** GPR56 which contains a mutation (L640R) is relevant to BFPP. aGPCRs, adhesion G protein-coupled receptors; AT1, alveolar type I epithelial cell; AT2, alveolar type II epithelial cell; BFPP, bilateral frontoparietal polymicrogyria; cDCs, conventional dendritic cells; CUB, C1r/C1s, Uegf, Bmp1; DS, dermatan sulfate; EGF, epidermal growth factor; FA, focal adhesion; PLL, pentraxin/laminin/neurexin/sex-hormone-binding-globulin-like; PNS, peripheral nervous system; RBC, red blood cell; SC, Schwann cell; SEA, sperm protein, enterokinase, agrin; SFTPD, surfactant protein D.

A missense variant in EMR2 expressed in mast cells, p. C492Y, may be a critical factor contributing to autosomal dominant vibratory urticaria (Fig. 2A). People with this variant often exhibit localized urticaria and degranulation of mast cells when subjected to dermal vibration. The investigators found that when cells adhered to DS or monoclonal anti-EMR2 antibody 2A1, the NTF of p. C492Y did not separate from the CTF in the absence of vibration. Nevertheless, after vibration, the NTF detached from the cell surface, resulting in a significant increase in degranulation.^39^ This suggests that the activation of EMR2 in mast cells is ligand-dependent, vibration-dependent, and dissociation-dependent.

Additional evidence for the idea that EMR2 is a mechanosensor comes from studies of its interaction with complement factor H-related protein 1 (FHR1). In monocytes, immobilized FHR1 or monoclonal antibody 2A1 activated EMR2. This signal was transmitted downward through the Gα_16_/PLCβ/MAPK/NF-κB axis, ultimately inducing macrophage-like differentiation as well as the production of the NLRP3 inflammasome and proinflammatory mediators. Interestingly, free or soluble FHR1 and 2A1 are ineffective, implying that the mechanical force generated by fixed ligand-mediated receptor binding and cross-linking plays a key role in this process.^40^, ^41^, ^42^ However, whether the activated EMR2-NTF in monocytes undergoes shedding as it does in mast cells and whether a similar EMR2/Gα_16_-mediated signaling cascade exists in other immune cells remains to be further investigated.

#### ADGRE5/CD97

ADGRE5/CD97 is in the same ADGRE subgroup as EMR2. Their five EGF domains are almost identical, differing by only six residues.^43^ CD97 is widely distributed on immunocytes, with the highest expression on myeloid cells, including monocytes, macrophages, and granulocytes. Moreover, epithelial cells, muscular cells, and fat cells express variable degrees of CD97.^44^ CD55, DS, Thy-1 (CD90), and integrins α5β1 and αvβ3 are known ligands of CD97.^45^, ^46^, ^47^, ^48^ The binding sites of different ligands to CD97 are distinct. For example, CD55 and DS bind to the EGF domains,^46^ whereas Thy-1 attaches to the stalk of the receptor.^47^ Recently, a study reported the cryogenic electron microscopy structures of CD97. In the inactive state, CD97 adopts a more compact conformation. The interaction between the GAIN domain and extracellular loops 1/2 is helpful for the tethered agonism of CD97. Upon activation, significant conformational changes occur on both the extracellular and intracellular sides of the receptor. This provides sufficient space to accommodate the Stachel sequence and downstream G proteins. Resolving the structure of CD97 lays the foundation for activation mechanism analysis of other aGPCRs.^49^

The spleen is known to be the largest secondary lymphoid organ, and adaptive immune responses will not be initiated properly if conventional dendritic cells in the spleen lose homeostasis. This homeostasis is maintained by the interaction between CD97 on conventional dendritic cells and CD55 on red blood cells (Fig. 2B). Co-culture of conventional dendritic cells with wild-type red blood cells revealed that whereas they expressed high levels of NTF^+^ CD97 in the absence of blood flow shear stress, this expression significantly decreased with shear stress. However, red blood cells lacking CD55 failed to regulate NTF^+^ CD97 regardless of whether shear stress was present or not. Consistent findings were obtained from *in vivo* experiments in mice. This suggests that blood flow shear stress and CD55 binding synergistically activate CD97, followed by NTF extraction and Stachel sequence exposure.^50^ Notably, the N-terminus of CD97 contains three to five EGF domains. The number of EGF domains determines the type of gene-spliced isoforms of CD97. Similarly, there are four short consensus repeat domains at the N-terminus of CD55. The EGF_1,2,5_ and short consensus repeat_1,2,3_ domains are the key determinants for CD55-CD97 interactions. The binding of these two molecules is roughly antiparallel, and this force-resisting shearing stretch structure may serve as the foundation for the receptor's mechanosensing.^51^

In addition to mediating the communication between conventional dendritic cells and red blood cells in the immune system, CD97-CD55 contact facilitates the adhesion of leukocytes and their retention at specific tissue sites. Karpus et al observed that the binding of CD55 to CD97 on leukocytes led to a decrease in CD97 and the release of CD97-NTF. This CD55-induced CD97 down-regulation required blood flow shear stress both *in vivo* and *in vitro*. Given that CD97 activates ERK, protein kinase B/Akt, or RhoA signaling pathways in prostate cancer cells, researchers further explored whether the binding of CD55 to CD97 could activate the same signaling pathways in leukocytes. Nevertheless, their interaction did not induce the activation of the above signaling.^52^ It is reasonable to speculate that the activation of CD97 in leukocytes probably triggers other signaling. However, it cannot be ruled out that CD97-CD55 only mediates intercellular adhesive contacts. Whether CD97-CD55 interaction initiates signaling transduction in leukocytes should be determined in the future.

Unlike the abovementioned function of directly sensing mechanical stimuli, CD97 may also act only as a participant in mechanical activities. Shear stress can activate protein kinase C/protein kinase D, which then rapidly induces phosphorylation of CD97 at the intracellular C-terminal PSD-95/discs-large/ZO-1 (PDZ)-binding motif. Although this phosphorylation does not lead to NTF shedding, it does interfere with the binding of CD97 to the PDZ domains of the scaffold protein DLG1. As a result, cells contract and lose intercellular contact at a faster rate due to poorer adhesion.^53^

#### ADGRG6/GPR126

ADGRG6/GPR126 belongs to the ADGRG subfamily. The structure of the ECD of this receptor is complex. It is composed of complement C1r/C1s, Uegf, Bmp1 (CUB); pentraxin; sperm protein, enterokinase, agrin (SEA); hormone receptor; and GAIN.^54^ GPR126 is expressed in multiple tissues and cells, especially in cells exposed to mechanical stimulation, including vascular endothelial cells, chondrocytes, urinary tract epithelial cells, and acinar secretory cells of the salivary gland.^55^ It has been shown that laminin-211, type IV collagen, and prion protein PrPC are effective extracellular binding partners for initiating receptor signaling.^56^, ^57^, ^58^

One of the best-studied functions of GPR126 is its involvement in myelination in the peripheral nervous system (Fig. 2C).^59^ During the formation of the peripheral nervous system, Schwann cells radially sort axons and then wrap around them to form myelin sheaths.^60^ Previously, the analysis of mutants in mice and zebrafish demonstrated that Gpr126 in Schwann cells drove myelin formation by raising the level of secondary messenger cAMP.^61^, ^62^, ^63^ However, researchers have recently turned their attention to the mechanism of GPR126 activation in Schwann cells. According to *in vitro* experiments, the interaction of laminin-211 with GPR126 controls receptor signaling via the Stachel sequence in a dissociation-dependent manner. Normally, a ligand binding to a receptor results in a single downstream phenotype. Nevertheless, contrary to common perception, the regulatory action of laminin-211 with GPR126 is dual and can either block or boost downstream signals. It may depend on whether mechanical forces are present. On the one hand, under static conditions, laminin-211 led to a reduction in cAMP levels by preventing the coupling of the receptor to G_s_ proteins; on the other hand, cAMP increased in cells transfected with GPR126 when exposed to vibration forces, and the addition of laminin-211 led to a more significant increase. This suggests a synergistic relationship between vibration and laminin-211. Consistent with the *in vitro* model, myelination was inseparable from Gpr126 agonism mediated by laminin-211 polymerization *in vivo*.^57^ Surprisingly, Paavola et al discovered that type IV collagen did not require the assistance of vibration forces to activate Gpr126 of zebrafish.^56^ A plausible explanation for this seeming contradiction is that the mechanical stimuli required for laminin-211 to activate GPR126 *in vivo* originate from the pulling force generated by polymerization-mediated receptor cross-linking. In the absence of cross-linking *in vitro*, this pulling force is replaced by vibration. Meanwhile, the mechanical stimuli required for receptor activation by type IV collagen come from the pushing force exerted on the receptor when the collagen resides on the cell layer.^64^ Hence, GPR126 needs mechanical forces and ligands to exert its functions in Schwann cells.

Interestingly, GPR126-NTF and GPR126-CTF have different functions at different stages of Schwann cell development. In the early stage, immature Schwann cells secrete laminin-211, which binds to GPR126-NTF. At this point, laminin, which has not undergone polymerization, inhibits downstream signaling by preventing the contact of the Stachel sequence with the 7TM domain. This process drives radial sorting in an NTF-dependent but CTF-independent manner. Later, laminin-211 polymerizes, which in turn causes the NTF and CTF to separate and the Stachel sequence to become accessible. This eventually facilitates an increase in cAMP and initiates myelin gene expression in promyelinating Schwann cells in a CTF-dependent manner.^57^ In addition, Gpr126-NTF modulates cardiac development independent of its CTF. Specifically, Gpr126-NTF is necessary for heart development, whereas Gpr126-CTF is not.^65^ These findings support the view that GPR126 has domain-specific functions.

It should be noted that there are other mechanisms of GPR126 activation in addition to the tethered agonism model as follows. i) The conformation of the ECD is altered to change the signal level. For example, Gpr126 lacking a splice insertion in the ECD has a closed conformation and basal signals, while Gpr126 with a splice insertion has a more dynamic and open conformation as well as enhanced signals. ii) Mutation of the calcium-binding site in the CUB domain results in defective signaling. iii) Due to the presence of a furin-cleavage site in the SEA domain (similar to the GPS in the GAIN domain), ligand binding or mechanical forces may modulate GPR126 activity by furin-dependent shedding.^54^ This illustrates the intricacy of GPR126 signal regulation.

#### ADGRF5/GPR116

ADGRF5/GPR116 is a member of the ADGRF subfamily. The N-terminus of ADGRF5/GPR116 contains two immunoglobulin-like repeats and a SEA domain. Its expression can be seen in tissues including the lung, kidney, liver, heart, and breast.^66^ GPR116 has long been categorized as an orphan receptor. It is only in recent years that an interaction between the NTF of this receptor and surfactant protein D (SFTPD), as well as fibronectin type III domain containing 4 (FNDC4), has been identified.^67^^,^^68^

GPR116 is abundantly expressed in alveolar type II epithelial cells, one of the primary physiological functions of which is to secrete and reuptake pulmonary surfactant (Fig. 2D).^69^ Surfactant is a mixture of lipids and proteins that prevents alveolar collapse by reducing surface tension during respiration.^70^ Gene knockout experiments in mice have confirmed that GPR116 maintains the homeostasis of surfactant *in vivo* by inhibiting its secretion.^71^^,^^72^ Knock-in mice expressing a non-cleavable mutant of Gpr116 shared the same pulmonary phenotype as Gpr116 knockout mice, *i.e.*, accumulation of surfactant and foamy alveolar macrophages in the distal airspaces.^73^ Moreover, *in vitro* experiments revealed that GPR116-CTF was able to activate the G_q/11_ signaling pathway in alveolar type II epithelial cells, while full-length GPR116 was not.^74^ These data suggest that GPR116 cannot be activated and exercise its function if the receptor does not undergo autoproteolysis and separation. As a putative ligand for GPR116, SFTPD can sense the amount of surfactant.^67^ Therefore, it can be hypothesized that the binding of SFTPD and the mechanical stretching of alveolar type II epithelial cells induced by continuous alveolar expansion and compression during the ventilatory cycle could control the activation of GPR116 *in vivo*.^74^ Nevertheless, there is no proof suggesting that SFTPD directly activates GPR116. The next phase of research could focus on exploring the ligands of GPR116 and validating the activation pattern of GPR116 in the alveolar lumen.

#### ADGRV1/GPR98

ADGRV1/GPR98 is also called very large G protein-coupled receptor-1 (VLGR1), which belongs to the ADGRV subfamily. Among its several isoforms, VLGR1b is known as the largest aGPCR with an extended ECD. However, whether its size is critical to its biological function remains to be further explored.^75^ The ECD of the receptor harbors multiple calcium exchanger β motifs, an epilepsy-associated repeat domain, and a pentraxin domain. VLGR1 is highly expressed in the central nervous system during the embryonic stage and mid-gestation, while its expression decreases in late gestation, which reflects the role of this protein in the development of the central nervous system.^76^^,^^77^ In addition, VLGR1 is involved in various functions, such as cell adhesion, cell cycle and gene regulation, ciliogenesis, and balancing Ca^2+^ homeostasis.^78^

Focal adhesions regulate cell migration and spreading by acting as signaling bridges between the extracellular matrix and the intracellular compartment.^79^^,^^80^ VLGR1 was found to be localized in focal adhesion protein complexes (Fig. 2E). In wild-type cells, fluid shear stress increased the number and length of focal adhesions and reduced cell spreading. However, in VLGR1-deficient cells, shear force had little effect on the number and length of focal adhesions and cell spreading. This suggests that VLGR1 acts as a mechanosensor by remodeling focal adhesions.^81^ Although the signaling network for this process is unclear, VLGR1, similar to most aGPCRs, can cleave at the GPS and split into two fragments.^82^ Furthermore, Knapp et al recently showed that full-length VLGR1 tended to couple to Gα_s_, while VLGR1-CTF tended to couple to Gα_i_. It is possible that shedding of the NTF triggers the binding of the Stachel peptide, consisting of 11 amino acids, to the CTF, which in turn induces the switch from Gα_s_-to Gα_i_-mediated signaling of VLGR1.^78^ Although these results imply the possibility that VLGR1 engages in mechanotransduction via a dissociation model, further studies are still needed to test this hypothesis. It will also be important to explore the link between the mechanosensory effects of VLGR1 and different cellular functions.

### Model of activation mainly through non-dissociation

#### ADGRG5/GPR114

Despite being a member of the same subfamily as GPR56 and GPR126, ADGRG5/GPR114 has a closer evolutionary relationship with GPR56. Currently, the NTF of GPR114 remains undefined. Transcripts of GPR114 can be seen in human eosinophils as well as in lymphocytes and monocytes/macrophages of rats and mice. So, this receptor may be mainly involved in the immune function of the organism.^83^ Few investigations have been conducted thus far on GPR114 ligands. However, the discovery of dihydromunduletone and 3-α-acetoxydihydrodeo-xygedunin as a small molecule antagonist and a partial agonist for GPR114, respectively, offers fresh perspectives for the development of aGPCR therapeutics in the future.^84^^,^^85^

Until recently, the question about the activation mechanism of GPR114 remained inconclusive. Previously, Wilde et al identified two natural isoforms of GPR114 in mice, which differed by the presence of a single amino acid (Q230) located at position 8 of the Stachel sequence. Position 8 served as a connector between the N-terminal activation part and the C-terminal orientation part. Furthermore, this amino acid was needed for the mechanosensory feature of the receptor because vibration-induced agonism was found in the full-length isoform (Q230) but not in the other isoform that lacks Q230 (ΔQ230) (Fig. 2F). The authors speculated that the agonistic part of the Stachel sequence had been prebound to 7TM and that the application of mechanical forces caused it to change into the active configuration.^17^ Based on the latest reported cryogenic electron microscopy structure of GPR114, Ping et al advanced a new hypothesis. According to their viewpoint, the Stachel sequence was presented as a β-sheet embedded in the GAIN domain, and activation stimuli induced the sequence to transform into an α-helical-bulge-β-sheet structure before moving to the binding pocket of the 7TM region.^24^ Notably, neither situation necessitates the removal of the NTF for receptor agonism. Consequently, GPR114 responds to mechanical forces through a non-dissociation model. In addition to identifying the exact mechanism of GPR114 activation, we also encourage researchers to pay attention to the exploration of ligands and the mechanosensitive role of this receptor in different cells, especially in immune cells.

#### ADGRL1-ADGRL3/CIRL1-CIRL3/Latrophilins

Latrophilins belong to the ADGRL subfamily. They have a long evolutionary history, and their genes can also be termed LPHN.^7^ The ECD of Latrophilins includes a rhamnose-binding lectin domain, olfactomedin domain, hormone receptor motif, and a GAIN domain.^86^ These receptors are highly expressed in nervous system tissues.^87^ In addition to acting as Ca^+^-independent receptors for α-latrotoxin, latrophilins bind to teneurin-2 (Lasso), neurexin, FLRT, and contactin-6 to participate in neuronal developmental processes.^88^^,^^89^

*Drosophila melanogaster* only has one homolog of latrophilins, called dCirl. In the larval stage, dCirl is expressed mainly in two different types of peripheral sensory neurons, the pentascolopidial chordotonal organs and the C4da neurons (Fig. 2G). As a low-threshold mechanoreceptor, the chordotonal organ controls drosophila's perception of touch, sound, and proprioception. dCirl subjected to gentle mechanical stimulation underwent a conformational change, which converted mechanical signals into a decrease in cAMP levels in a manner dependent on intact TA but not on NTF-CTF separation. The reduction in cAMP amplified signal transduction, which in turn enhanced the receptor response to this harmless stimulus. It is worth mentioning that chordotonal organs also contain transient receptor potential channels, whose subunits NompC and Nanchung can interact with dCirl, allowing ion flux to be regulated by aGPCR activity. This implies a synergism between metabolic receptor signaling and ionotropic signaling during sensory formation.^90^^,^^91^

In contrast, C4da neurons are high-threshold nociceptive receptors. Strong pain stimulation activates dCirl and reduces cAMP levels by inhibiting the activity of adenylate cyclase through G_i/o_, which does not require the involvement of the Stachel sequence. As a result, the responsiveness of nociceptive receptors to harmful stimuli is weakened. This finding shows that dCirl plays an anti-damage role in C4da neurons. cAMP decline leads to a bidirectional regulatory effect probably because the secondary messenger cascade acts on different ion channels.^92^ At present, the TA-independent activation mechanism is not understood, nor is it clear whether the anti-injury and analgesic effects of dCirl are applicable to mammals. Future research should focus on the mechanosensory role of latrophilins in mammals.

### Model of activation either through dissociation or non-dissociation

#### ADGRG1/GPR56

ADGRG1/GPR56 is a member of the ADGRG subfamily. Its ECD consists of a GAIN domain and a pentraxin/laminin/neurexin/sex-hormone-binding-globulin-like domain.^93^ This receptor can be found in the brain, heart, thyroid, kidney, testis, and skeletal muscle.^94^ Several ligands have been identified, including collagen III, transglutaminase 2, tetraspanins CD9/CD81, progastrin, and phosphatidylserine.^95^^,^^96^ Researchers have previously used biochemical and cell-based methods to investigate the activation mechanism of GPR56. They discovered that isolating the NTF from full-length GPR56 significantly enhanced G protein activation while removing individual amino acids from the N-terminus of the 7TM domain sequentially reduced the receptor activity to zero. These findings indicated a tethered agonist and a dissociation hypothesis for GPR56 activation.^18^

Shear flow is produced by circulating platelets rolling along collagen at the site of vascular injury. Recently, it has been shown that immobilized collagen and blood flow-induced shear force can stimulate GPR56 on platelets. This promotes the separation of the NTF from the CTF and ultimately leads to morphological changes in platelets as well as the formation of adhesions and plugs via Gα_13_ signaling. In addition, *Gpr56*^*−/−*^ mice have a protracted bleeding duration, impaired platelet plug formation, and delayed thrombus occlusion.^97^ Therefore, GPR56 is crucial for hemostasis.

Mutations in GPR56 are related to a human brain malformation called bilateral frontoparietal polymicrogyria, which is commonly accompanied by disturbed cortical lamination (Fig. 2H).^98^ According to Luo et al, collagen III serves as the main ligand of GPR56 in neurons, and their connection regulates normal cortical lamination by inhibiting neuronal migration.^99^ In response to collagen III stimulation, the NTF of wild-type GPR56 was released from the CTF, which caused the CTF to translocate to lipid rafts, and this triggered a downstream Gα_12/13_-RhoA signaling cascade.^100^^,^^101^ Surprisingly, GPR56 with a bilateral frontoparietal polymicrogyria-related mutation (L640R) failed to induce the activation of RhoA signaling, despite the separation of the NTF from the CTF.^100^ Accordingly, the pathogenesis of bilateral frontoparietal polymicrogyria may be that the mutation prevents receptor coupling to G proteins, thereby abrogating the normal physiological function of GPR56. Although no clear mechanical forces have been observed throughout the signal transduction process, there is evidence that physical forces are associated with the development of the nervous system.^102^ Furthermore, macromolecular fibrils assembled from triple-helix collagen III monomers may also have cross-linking effects on the receptor, thus providing the mechanical forces required for activation.^103^

Apart from its role in the developing brain, GPR56 is also involved in the regulation of central nervous system myelination by oligodendrocyte lineage cells. GPR56 on oligodendrocyte precursor cells forms a tripartite signaling complex with extracellular laminin-111 and microglia-derived transglutaminase 2, which contributes to oligodendrocyte precursor cell proliferation and myelin formation via Gα_12/13_ and RhoA signaling. The presence of laminin, receptor cleavage, and NTF shedding are indispensable to this process.^101^^,^^104^ Notably, transglutaminase 2 can cross-link laminin.^105^ Consequently, transglutaminase 2 binding alone does not work, probably due to the lack of traction generated by cross-linking, which could lead to the separation of the NTF from the CTF.

Similarly, receptor cross-linking mediated by fixed CG4 monoclonal antibody (GPR56-specific) but not soluble CG4 can induce GPR56 activation in melanoma cells with the help of the tetraspanins CD9 and CD81. The CD9/CD81-GPR56 complex may adopt a unique conformation that promotes NTF shedding upon CG4 binding.^106^

GPR56 can also modulate mechanical load-induced muscle hypertrophy. When exposed to overload stimuli, wild-type mice showed muscle hypertrophy and increased expression of Gpr56 and its ligand type III collagen; meanwhile, *Gpr56*^*−/−*^ mice presented attenuated hypertrophy and associated signals. Additionally, muscle regeneration was not achievable in *Gpr56*^*−/−*^ mice after injury.^107^^,^^108^ These data indicate that mechanical forces engage in the activation of GPR56 in signaling pathways associated with muscle hypertrophy.

Except for the dissociation-dependent manner, GPR56 also participates in physiological and pathological events in a dissociation-independent mechanism. The synthetic protein ligands targeted to GPR56 ECD induced a direct interaction of the ECD with 7TM, which changed signaling.^22^ However, we still do not know the effect of synthetic ligands on cellular function and whether there are natural extracellular binding partners that activate GPR56 in a dissociation-independent manner.

## Mechanosensitive aGPCRs and diseases

**Figure 3:**
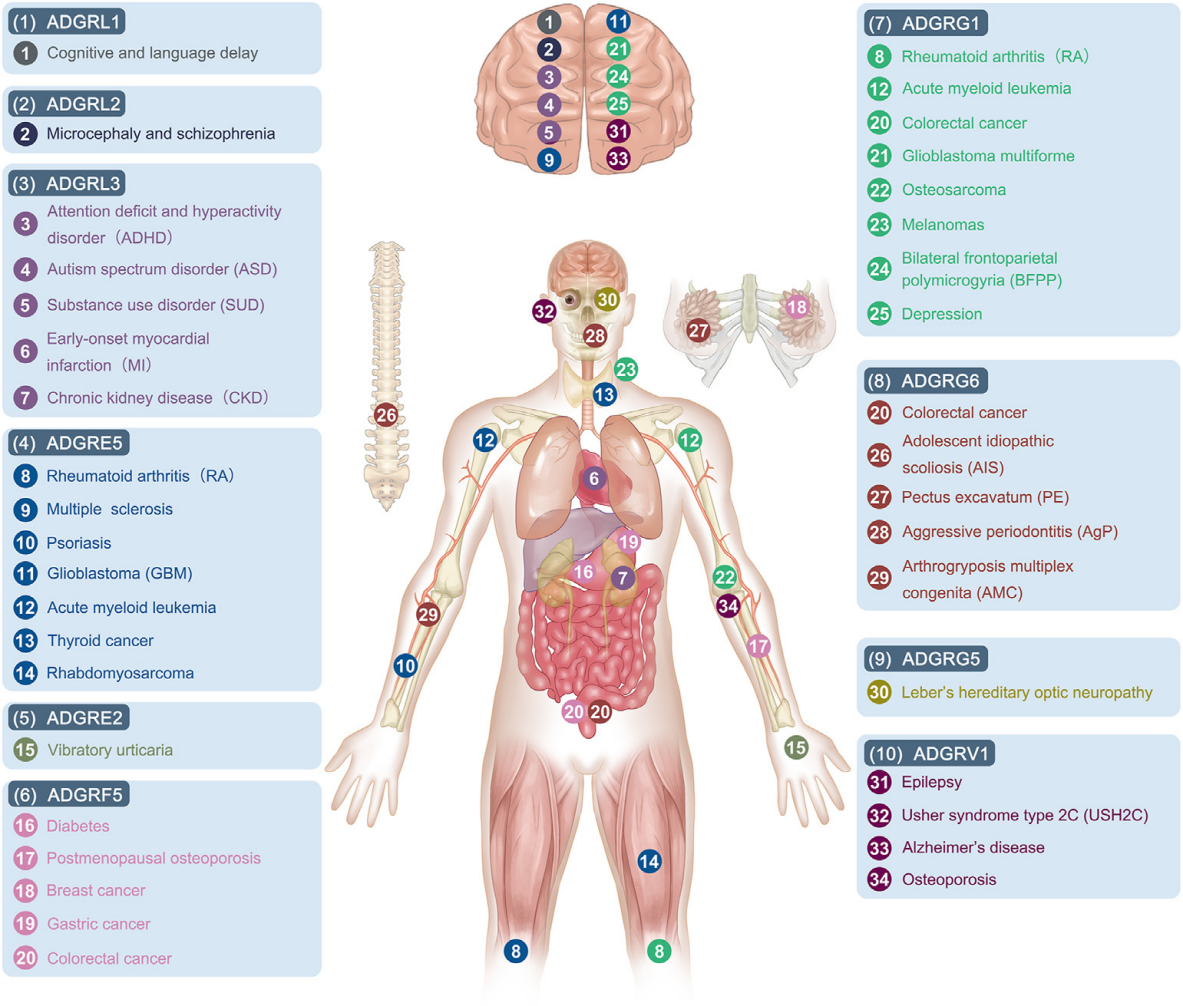
Diseases associated with mechanosensitive adhesion G protein-coupled receptors (aGPCRs). Different colors are used to distinguish diseases related to different receptors.

In the above section, we discussed that 10 mechanosensitive aGPCRs are engaged in different physiological and pathological processes in the presence of mechanical stimuli. Moreover, these receptors have been shown to be associated with many other diseases, but whether mechanical forces are involved in those pathological processes is still unknown. In this section, we present the relevance of 10 mechanosensitive aGPCRs to these diseases by classifying these receptors into different subfamilies. Future studies are needed to further explore the mechanotransduction processes in them (Fig. 3).

### ADGRL subfamily

As mentioned above, latrophilins are abundantly expressed in neural tissues,^87^ so it is not surprising that these receptors are implicated in various neurological disorders. For example, microdeletions of ADGRL1 may be a potential cause of cognitive and language delay,^109^ while ADGRL2 variants are related to microcephaly and schizophrenia.^110^^,^^111^ In contrast, single nucleotide polymorphisms associated with ADGRL3 are thought to increase susceptibility to attention deficit and hyperactivity disorder, autism spectrum disorder, and substance use disorder.^112^, ^113^, ^114^, ^115^ In addition to these neurological diseases, exome-wide association studies have found that genetic variation in ADGRL3 has a significant association with early-onset myocardial infarction and chronic kidney disease in Japanese individuals.^116^

### ADGRE subfamily

ADGRE5 is widely expressed in immune cells and is related to several immune diseases. Soluble ADGRE5 is known to be released under inflammatory conditions.^117^ Previous studies have found elevated expression of soluble ADGRE5 in the synovial fluid of patients with rheumatoid arthritis.^118^ Later, Hoek et al demonstrated in an experimental mouse model of rheumatoid arthritis that the interaction between ADGRE5 and its ligand, CD55, contributed to the development of arthritis. The absence of either molecule led to the amelioration of inflammation.^119^ This highlights the catalytic role of ADGRE5 in rheumatoid arthritis development. Notably, the ADGRE5-CD55 pair is also involved in the inflammatory process of multiple sclerosis.^120^ In patients with psoriasis, the binding of ADGRE5, which has enhanced expression on leukocytes, to Thy-1 on activated vascular endothelial cells mediates the adhesion interaction between the two cells. This interaction is essential for the accumulation of leukocytes at the inflammatory site.^47^ Moreover, another member of this subfamily, ADGRE2, plays a role in the progression of immune disorders. Its relevance to the pathogenesis of vibratory urticaria has been described in detail in the previous section.^39^

Multiple studies have shown that ADGRE5 is an overexpressed tumor antigen in several cancer types and is associated with tumor migration, invasion, and metastasis. For example, ADGRE5 knockdown in glioblastoma cell lines reduces invasion and migration, and the expression of this protein is inversely correlated with survival.^121^ Up-regulation of ADGRE5 expression in hepatocellular carcinoma promotes tumor metastasis, which is mediated by matrix metalloproteinase 2/9 secretion as a result of the interaction between the receptor and G protein-coupled receptor kinase 6.^122^ Similarly, excessive ADGRE5 expression in acute myeloid leukemia facilitates the proliferation and survival of leukemic blasts, maintains their undifferentiated state, and thus contributes to poor prognosis.^123^ Other studies have also indicated that high levels of ADGRE5 are positively correlated with the invasion and metastasis of tumors such as thyroid cancer and rhabdomyosarcoma.^124^^,^^125^ Recently, Ward et al proposed that ADGRE5 serves as a platelet receptor on tumor cells and that it promotes tumor invasion and metastasis by stimulating platelet activation.^126^ These findings support the use of ADGRE5 as a therapeutic target to reduce tumor metastatic spread.

### ADGRF subfamily

ADGRF5 controls systemic glucose homeostasis in white adipose tissue by working as a receptor for soluble FNDC4. On the one hand, the FNDC4-GPR116 axis is damaged in patients with diabetes; on the other hand, injection of recombinant FcsFNDC4 corrected hyperglycemia in prediabetic mice by inducing the GPR116-G_s_-cAMP signaling pathway in adipocytes.^68^ Additionally, ADGRF5 may play a role in postmenopausal osteoporosis by regulating the NF-κB signaling pathway.^127^

With regard to cancer, Tang et al identified ADGRF5 as a regulator of breast cancer metastasis through gene expression and functional screening. The investigators found that low levels of ADGRF5 in highly metastatic cancer cells inhibited cell migration and invasion *in vitro*, whereas ectopic ADGRF5 expression in low metastatic cells had the opposite effect. Further studies have shown that the role of this receptor in breast cancer is mediated by the Gα_q_-p63RhoGEF-Rho GTPase signaling pathway. These results were confirmed in human breast cancer clinical samples which showed that ADGRF5 expression was significantly related to metastasis of breast tumors and poor prognosis.^128^ Other studies have shown that this receptor can also be used to predict poor prognosis in patients with gastric and colorectal cancers.^129^^,^^130^

### ADGRG subfamily

Similar to several subfamilies mentioned above, members of the ADGRG subfamily are also associated with cancer. Both ADGRG1 and ADGRG6 are involved in the progression of colorectal cancer. The proliferation of colorectal cancer cells mediated by ADGRG1 may require progesterone binding.^131^^,^^132^ High expression of ADGRG1 also causes poor prognosis in patients with glioblastoma multiforme, acute myeloid leukemia, and osteosarcoma.^133^, ^134^, ^135^, ^136^ Notably, unlike other tumors, ADGRG1 is significantly down-regulated in highly metastatic human melanomas. This receptor might interact with the extracellular matrix protein transglutaminase 2 to limit the growth and spread of melanoma.^137^

Mutations in ADGRG6 are factors in some musculoskeletal disorders, including adolescent idiopathic scoliosis, pectus excavatum, arthrogryposis multiplex congenita, and periodontitis. Several genome-wide association studies based on ethnically diverse populations have found that the ADGRG6 single nucleotide polymorphism rs6570507 is significantly related to adolescent idiopathic scoliosis susceptibility.^138^, ^139^, ^140^, ^141^ Karner et al also confirmed that Adgrg6 is a genetic factor important for the etiology of adolescent idiopathic scoliosis and pectus excavatum.^142^ In humans, a missense substitution of ADGRG6 impairs autoproteolysis of the receptor, which results in severe arthrogryposis multiplex congenita.^143^ Furthermore, in the Japanese population, the ADGRG6 single nucleotide polymorphism rs536714306 may have an association with aggressive periodontitis. Under normal conditions, ADGRG6 may maintain the homeostasis of human periodontal ligament tissues by up-regulating the cellular differentiation of human periodontal ligament cells; however, mutants can impair this up-regulation, break homeostasis, and lead to the development of aggressive periodontitis.^144^

Apart from tumors and musculoskeletal disorders, members of this subfamily have been related to other disorders. For example, in the brain, a mutation in ADGRG1 contributes to bilateral frontoparietal polymicrogyria.^98^ Down-regulation of ADGRG1 mediates depression in humans or depression-like behaviors in mice.^145^ In the immune system, soluble ADGRG1, which is elevated in the serum of rheumatoid arthritis patients, can be used as a biomarker for rheumatoid arthritis.^146^ A recent genome-wide association study also suggested that mutations in ADGRG5, another member of the ADGRG subfamily, might be involved in the occurrence of Leber's hereditary optic neuropathy.^147^

### ADGRV subfamily

Mutations in ADGRV1 often cause the following two disorders: epilepsy and Usher syndrome type 2C.^148^, ^149^, ^150^ The occurrence of epilepsy may be associated with defective cell migration caused by ADGRV1 dysfunction.^81^ Usher syndrome type 2C mostly affects inner ear hair cells and photoreceptor cells, and its primary symptoms include sensorineural hearing loss and retinitis pigmentosa.^151^^,^^152^ ADGRV1 is present not only in the ankle links between the stereocilia of the inner ear hair cells but also in the fibrous links of photoreceptor cells. Adgrv1-deficient mice showed disrupted fibrous links in both types of cells and exhibited auditory impairment.^153^^,^^154^ This confirms the role of the ADGRV1 mutant in the pathogenesis of Usher syndrome type 2C.

Recently, Knapp et al identified ADGRV1 in Alzheimer's disease-related protein complexes, indicating a link between this receptor and the illness.^78^ In addition, the ADGRV1 gene is thought to be involved in the regulation of bone mineral density and is associated with susceptibility to osteoporosis.^155^

## Conclusions and perspectives

We provide a description of the structural characteristics, signal transmission, activation modes, and related diseases of 10 known mechanosensitive aGPCRs. The presence or absence of mechanical stimuli and ligands, as well as the ability of autoproteolytic cleavage, affect the agonism and downstream signaling of these mechanoreceptors. Although several studies have elucidated the mechanisms by which some aGPCRs regulate mechanotransduction, these mechanisms remain elusive for all 33 aGPCRs. Therefore, future research should not only explore more potential mechanosensitive aGPCRs but also shed light on the specific activation conditions and physiological significance of these receptors in different tissues. Finally, these aGPCRs offer great potential as drug targets given the diversity of disorders in which they are involved. In the future, it will be important to develop novel targeted therapeutic approaches.

## Author contributions

S.S. performed the literature research and drafted the manuscript. W.W. contributed to the conception and design and critically revised the manuscript. Both authors read and approved the final manuscript.

## Funding

This work was supported by grants from the 10.13039/501100003787Natural Science Foundation of Hebei Province (China) (No. H2020206226), Hebei Province Science and Technology Support Program (China) (No. 18277756D), the Science and Technology Research Project of Hebei Higher Education Institutions (China) (No. ZD2022010), and High-level talent funding project of Hebei (China) (No. 20231141) to W.W.

## Conflict of interests

The authors declared no competing interests.
